# Alexithymia mediates the relationship between interoceptive sensibility and anxiety

**DOI:** 10.1371/journal.pone.0203212

**Published:** 2018-09-13

**Authors:** Eleanor R. Palser, Clare E. Palmer, Alejandro Galvez-Pol, Ricci Hannah, Aikaterini Fotopoulou, James M. Kilner

**Affiliations:** 1 Psychology and Language Sciences, University College London, London, United Kingdom; 2 Institute of Neurology, London, University College London, United Kingdom; Anglia Ruskin University, UNITED KINGDOM

## Abstract

A number of empirical and theoretical reports link altered interoceptive processing to anxiety. However, the mechanistic understanding of the relationship between the two remains poor. We propose that a heightened sensibility for interoceptive signals, combined with a difficulty in attributing these sensations to emotions, increases an individual’s vulnerability to anxiety. In order to investigate this, a large sample of general population adults were recruited and completed self-report measures of interoceptive sensibility, trait anxiety and alexithymia. Results confirmed that the positive association between interoceptive sensibility and trait anxiety was partially mediated by alexithymia, such that those most at risk for clinically significant levels of trait anxiety have both significantly higher levels of interoceptive sensibility and alexithymia. A subsequent factor analysis confirmed the independence of the three measures. Altered interoceptive processing in combination with alexithymia, increased the risk for anxiety above and beyond altered interoceptive processing alone. We suggest that a heightened sensibility for interoceptive signals, combined with a difficulty in attributing these sensations to emotions, leaves these sensations vulnerable to catastrophizing interpretation. Interventions that target the attribution of bodily sensations may prove valuable in reducing anxiety.

## Introduction

Anxiety disorders represent the most prevalent mental disorder worldwide [1], yet an understanding of their underlying psychological causes remains elusive. One promising research avenue concerns altered interoceptive processing. Interoception is defined as the detection of the physiological state of the body [2]. The accurate detection and response to these physiological states is crucial for maintaining homeostasis [2]. Interoceptive signals are conveyed via small diameter afferent fibers originating in the tissues and organs of the body, terminating in the posterior insula [2,3]. Interoception has been theoretically [4, 5] and empirically (e.g. [6]), linked to anxiety. Specifically, the misattribution of bodily sensations has been proposed as the key interoceptive component of anxiety [7], however the psychological and neurological mechanisms of this relationship are not well understood. Here, we propose that the reason these signals are misattributed is the presence of the sub-clinical construct alexithymia: a difficulty in identifying and describing one’s own emotions [8].

Interoceptive processing has been measured using a number of methods, for example self-report questionnaires (e.g. Autonomic Perception Questionnaire [9]; Body Perception Questionnaire [10]) and objective behavioral paradigms, which are most commonly heartbeat detection tasks [11, 12]. There has been some inconsistency in the literature in terms of the terminology used to describe the seemingly independent facets recorded using these different methods. In order to address this issue, a three-dimensional construct of interoception has been proposed [13, 14]. Paradigms that objectively quantify participants’ performance in detecting internal events represent measures of ‘interoceptive accuracy’ (or ‘sensitivity’ in [13]). Questionnaire based self-report measures of interoceptive processing, and average confidence judgements on objective tasks, are thought to quantify an individual’s perceived awareness of interoceptive signals and are termed ‘interoceptive sensibility’. Finally, the trial-wise correspondence between performance on objective measures and confidence judgements, referred to as ‘interoceptive awareness’, is thought to reflect an individual’s metacognitive insight into their interoceptive ability.

The inconsistent and interchangeable use of terminology has contributed to some of the confusion in the literature on interoception and anxiety. However, there have also been mixed results within a single domain of interoceptive processing. For example, in some studies, enhanced interoceptive accuracy, measured using a heartbeat counting task [11], is over-represented amongst anxiety patients [15, 16, 17]. However, in other studies no relationship has been found (e.g. [18]). Some studies have reported the inverse relationship, with higher anxiety symptoms in those with poorer interoceptive accuracy [19]. Varying levels of anxiety across samples and different measurement protocols likely play some role in these divergent findings, but it is also possible that a third construct mediates the relationship between altered interoception and anxiety.

When the perception of somatic sensations is measured using self-report questionnaires–termed interoceptive sensibility in Garfinkel & Critchley’s [13] formulation of interoceptive processing–patients with anxiety disorders tend to report a hypersensitivity to these sensations [20, 21, 22, 23]. These questionnaire measures (e.g. Body Sensations Questionnaire [24]; Body Vigilance Scale [25]) ask participants to rate how aware they are of a wide range of bodily states.

It has been proposed that individuals with high anxiety have a reduced signal-to-noise ratio of afferent interoceptive information, such that it is more difficult to differentiate between meaningful interoceptive signals that may flag pleasant or aversive consequences, and constant low-level fluctuations in the interoceptive state. This is paired with an altered belief system such that these individuals are biased to believe these low-level fluctuations are meaningful and aversive [4, 5]. Under this model, incorrect inferences about the causes of these interoceptive prediction error signals may directly contribute to the symptoms of anxiety disorder (e.g. changes in affect, worrisome thoughts and avoidance behaviors).

Here, we propose that one of the reasons interoceptive prediction error signals are misattributed, often to aversive causes, is due to the presence of alexithymia. Alexithymia, a specific difficulty in identifying and describing one’s own emotions [8] is found in approximately 13% of the general population [26]. A number of studies have found an association between alexithymia and anxiety traits in nonclinical samples (e.g. [27, 28]) and an association between alexithymia and altered interoceptive processing (e.g. [29]). Alexithymia is seen in much higher rates in individuals diagnosed with an autism spectrum disorder [30], with comorbidity of around 50% frequently reported [31, 32]. Both interoceptive differences and anxiety are also common in this population [33, 34], suggesting an association between these constructs. Indeed, a co-occurrence of comorbid anxiety, alexithymia and interoceptive differences are present in a number of psychological conditions, including eating and feeding disorders [35, 36, 37, 38, 39, 40, 41], substance dependence [42, 43, 44] and depression [27, 45, 46].

In the domain of interoceptive accuracy, a number of reports have found reduced performance in detecting and using interoceptive signals in individuals with alexithymia ([47, 48], although see [49, 50] for recent null results). In the domain of interoceptive sensibility, by contrast, there is evidence to suggest that alexithymics are hypersensitive to interoceptive sensations [51, 52, 53]. Divergent findings across interoceptive domains are not unusual either in normative [14], or clinical populations [33, 34]. Indeed, correspondence across dimensions is generally only seen in individuals with very good interoceptive accuracy. Taken together, these results suggest an interoceptive profile in alexithymia of reduced interoceptive accuracy and heightened interoceptive sensibility, similar to that previously reported in autism [33, 34].

Here, we propose that heightened interoceptive sensibility in combination with a difficulty attributing these signals to emotional states, operationalized here as alexithymia, predisposes an individual to anxiety-related characteristics. This elevates their risk for anxiety over and above altered interoceptive processing or alexithymia alone. The hereto mixed results in the interoceptive accuracy literature suggests that it may be the perceived awareness of interoceptive signals that is most robustly related to anxiety, not objective ability to accurately detect interoceptive signals. As such, we measure interoceptive sensibility here using a self-report questionnaire measure. As in previous research (e.g. [27, 28]), we probe anxiety traits in the general population to inform our understanding of the clinical disorder. We therefore test the hypothesis that the relationship between self-report interoceptive sensibility and anxiety is mediated by alexithymia.

## Methods

### Participants

A total of 426 adults participated as part of a science engagement project at a music festival. Ethical approval was granted by University College London Ethics Committee, all procedures were conducted in accordance with the Declaration of Helsinki and informed written consent was obtained from all. Any participants either missing questionnaire or demographic data were excluded, leaving a final sample of 384. Ages ranged between 16 and 65 years (mean = 33.49, SD = 13.87), and 255 were female (66%).

### Measures

Participants completed both State and Trait forms of the State Trait Anxiety Inventory (STAI [54]), the Awareness subsection of Porges Body Perception Questionnaire (BPQ [10]) and the Toronto Alexithymia Scale (TAS-20 [55]). A simplified version of the BPQ, developed in-house (S1 Text; [33]), was used to measure interoceptive sensibility. This version contains 39 items describing various sensations experienced in the body, and participants rate how often they experience each on a five-point scale of 1 (‘Never’) to 5 (‘Always’). The TAS-20 contains 20 items which are scored by participants on a five-point scale from 1 (‘Strongly Disagree’) to 5 (‘Strongly Agree’). The STAI contains two forms, the STAI-STATE asks participants to score twenty items on a four-point scale from 1 (‘Not at all’) to 4 (‘Very much so’) based on how well each item best describes them in the present moment. Similarly, the STAI-TRAIT asks participants to score twenty items on the same scale as the STAI-STATE based on how they generally feel. As we were primarily interested in dispositional anxiety traits, we only included anxiety data from the STAI-TRAIT in the present analysis.

### Procedure

The experiment was explained to participants by one of the researchers and informed consent was obtained from all. Participants were given a questionnaire booklet to complete, containing all three measures.

### Data analysis

Preliminary analyses assessed the influence of demographic characteristics (age, sex) on the variables under investigation using independent t-tests (sex) and Pearson correlations (age). Then, relative contributions of interoceptive sensibility and alexithymia to anxiety scores were assessed using simple and multiple regression analysis, using the four-step method of testing for mediation proposed by Baron and Kenny [56]. Thirdly, factor analysis using Varimax (orthogonal) rotation was used to assess whether any associations found between the variables of interest were a result of similarity between the measures.

## Results

Of the final sample of 384, 66 participants were above the cut-off for alexithymia (17.19%), a little higher than the previous 13% seen in a general population sample [26] using the same cut-off of a total score greater than 61. Trait anxiety was significantly higher in women (mean = 44.94, SD = 10.69) than men (mean = 41.60, SD = 10.35) [*t*(382) = 2.93, *p* = 0.004, *d* = 0.32]. Alexithymia was significantly higher in men (mean = 51.88, SD = 9.07) than women (mean = 48.48, SD = 10.69) [*t*(297.28) = 3.268, *p* = 0.001, *d* = 0.34]. Interoceptive sensibility was significantly higher in women (mean = 2.59, SD = 0.51) than men (mean = 2.43, SD = 0.53) [*t*(382) = 2.81, *p* = 0.005, *d* = 0.30]. All three measures were found to significantly decrease with age, including trait anxiety [*r* = -0.30, *p*<0.001], alexithymia [*r* = -0.24, *p*<0.001] and interoceptive sensibility [*r* = -0.26, *p*<0.001] [df = 382]. Sex and age were therefore entered as control variables in subsequent analyses.

**Fig 1:**
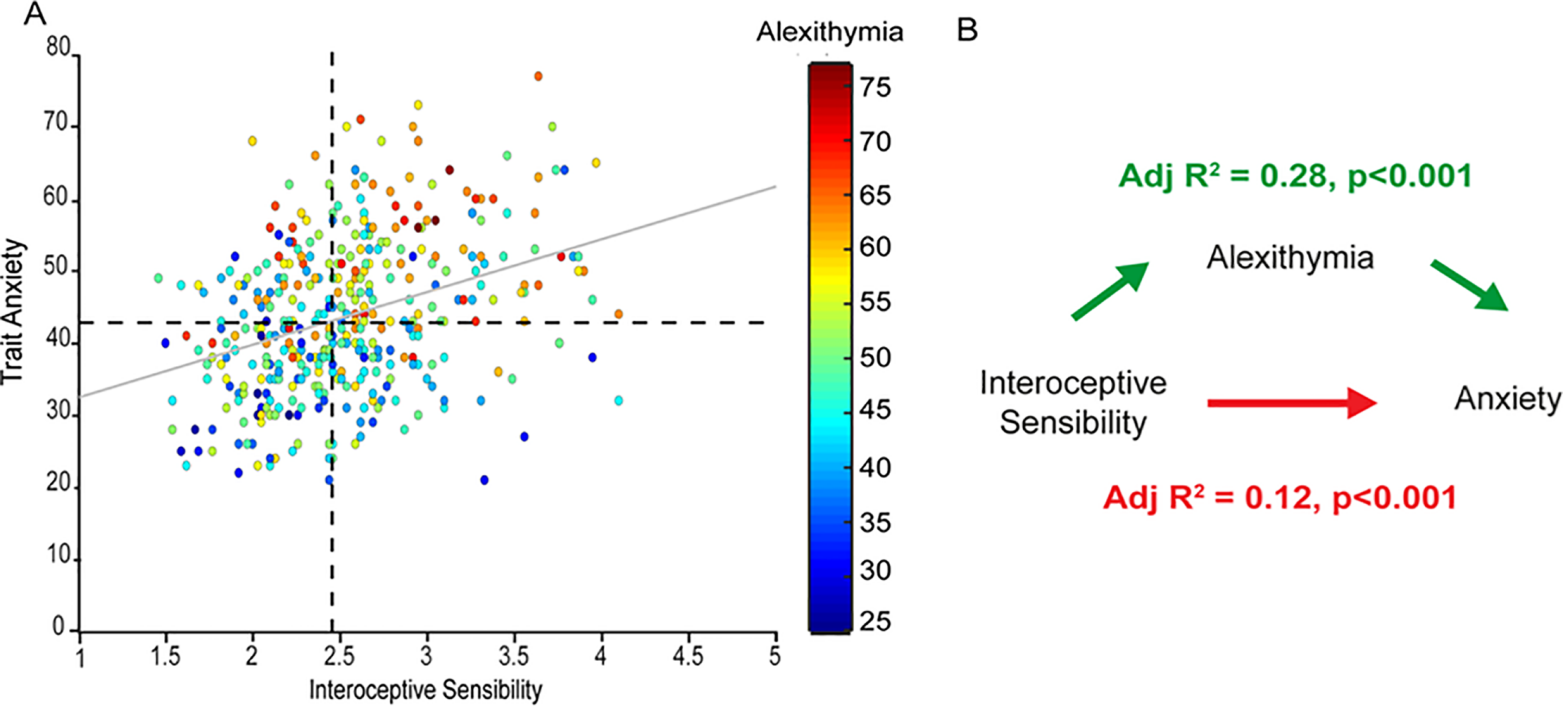
The positive association between interoceptive sensibility and trait anxiety is partially mediated by alexithymia, such that those most at risk for clinically significant levels of trait anxiety have both a propensity for high interoceptive sensibility and high levels of alexithymia (represented in the upper right quadrant of the plot [Panel A]). Including both alexithymia and interoceptive sensibility in the model explains more variance than simply including interoceptive sensibility alone [Panel B].

A four-step regression analysis was used test our hypothesis that alexithymia mediates the relationship between interoceptive sensibility and anxiety. Firstly, simple regression confirmed that interoceptive sensibility significantly predicted trait anxiety [*β* = 0.36, *p*<0.001], interoceptive sensibility predicted alexithymia [*β* = 0.20, *p*<0.001], and alexithymia predicted trait anxiety [*β* = 0.40, *p*<0.001]. A multiple regression with age, sex, interoceptive sensibility and alexithymia as predictors revealed both interoceptive sensibility [*β* = 0.23, *p*<0.001] and alexithymia [*β* = 0.34, *p*<0.001] to be significant predictors of trait anxiety, explaining more of the variance in anxiety scores [Adjusted R^2^ = 0.28] than either interoceptive sensibility or alexithymia alone [Adjusted R^2^ = 0.12 and 0.16, respectively]. Those with high interoceptive sensibility and alexithymia most likely also had high trait anxiety (Fig 1).

As Kolmogorov-Smirnov tests indicated that responses to individual items within the measures were not normally distributed (*p*<0.001), a factor analysis using the principal axis factors method of extraction with Varimax (orthogonal) rotation was used to verify that the associations observed were not a result of similarity between measures. The Kaiser-Meyer-Olkin measure of adequate sample size (KMO = 0.86) and the Bartlett’s test of sphericity (*p*<0.001) returned satisfactory values to proceed. The scree test suggested there were three separate factors in the data, representing the three separate questionnaires (see Factors 1–3, S1 Table). However, these three factors only cumulatively explained 27.91% of the variance. Using the criterion of retaining all factors with an Eigenvalue of greater than 1.0, indicated 20 separate factors in the data. Retaining these 20 factors, a component matrix was obtained after 22 iterations, suggesting that the measures of interoceptive sensitivity, alexithymia and trait anxiety all loaded onto separate factors (see S1 Table).

## Discussion

Our hypothesis that alexithymia plays a mediating role between interoceptive sensitivity and anxiety traits was supported—individuals with both high interoceptive sensibility and alexithymia scores are the most likely to report clinically significant levels of anxiety. Factor analysis confirmed that this was not the result of overly similar items across the questionnaires. These data provide support for the theory that the perception and misattribution of bodily sensations is a key component of anxiety [7], highlighting the additive role that alexithymia plays in this relationship. Indeed, individuals with anxiety may struggle to attribute bodily sensations accurately due to the presence of alexithymia.

The relationship between anxiety and interoception is clearly complicated, as demonstrated by the mixed results to date. Some studies find heightened interoceptive accuracy in anxiety [15, 16, 17], while others find it to be reduced [19]. Anxiety disorders represent a diverse category of conditions including panic disorder, social anxiety disorder, generalized anxiety disorder and specific phobias [57]. As such, it is likely that heterogeneous samples with diverse symptoms, as well as varying levels of symptom severity, contribute to the divergent findings to date. This is in addition to the role of different measurement protocols.

However, while the present findings relate only to the domain of interoceptive sensibility, it is worth considering that the inconsistencies in the literature may also be explained by the neglect of alexithymia as a mediating factor. Indeed, it was recently reported that alexithymia may fulfil this role in the relationship between interoceptive processing and autism [48, 58], with the relationship between autism and reduced interoceptive accuracy disappearing after controlling for alexithymia (however, see [59]).

Doubt is shed on this hypothesis however, by two recent publications, which find no association between alexithymia and interoceptive accuracy in both the general population, and autism [49, 50]. It was also recently reported that the relationship between interoceptive accuracy and alexithymia is only significant when a number of potential psychological and physiological confounds are accounted for [60]. These 11 confound variables include age, sex, time perception, anxiety, depression, body mass index (BMI), mean heart rate, knowledge about heart rate, systolic blood pressure, and heart rate variability [60]. The absence of measures of many of these confound variables in both [49] and [50] may account of these null findings.

Within the domain of interoceptive sensibility, the majority of previous studies find heightened perceived awareness of interoceptive signals in alexithymia [51, 52, 53]. Mehling [61] highlights the importance of differentiating maladaptive anxiety-driven attention to interoceptive sensations and a more adaptive, mindful attention style. The Multidimensional Assessment of Interoceptive Awareness (MAIA [62]) was developed as a measure of adaptive interoceptive sensibility and has been found to be negatively associated with alexithymia [50, 63]. Complimentary preliminary findings, of an association between interoceptive confusion and alexithymia, using an Interoceptive Confusion Questionnaire, have also been reported [64]. Taken together with the more traditional questionnaire measures of interoceptive sensibility, the picture in alexithymia appears to be of an anxiety-related, maladaptive interoceptive style.

This study provides empirical evidence to support current theories that describe an inability to cognitively appraise and affectively label interoceptive signals as a key component in the development of anxiety. There is debate in the existing literature as to whether anxiety sufferers more or less accurately perceive interoceptive signals, pay greater attention to them, or simply attribute these sensations to more catastrophic interpretations. The present findings do not provide information about the accuracy with which high-anxiety individuals perceive interoceptive signals, as this was not objectively measured. What is clear, however, is that these individuals report noticing bodily states more often than those with low anxiety. This is suggestive of an attentional style that focusses on interoceptive sensations. The present results also suggest this is combined with a difficulty in accurately interpreting and labelling these sensations, which fits well with the idea that these sensations may be misattributed as troubling or dangerous [65].

Theoretically, it has been suggested that anxiety is associated with altered inference about the causes of interoceptive signals [4]. Contributing to this is a reduced signal to noise ratio for afferent interoceptive information, and altered beliefs about, and heightened anticipation of, the aversive consequences of interoceptive states [4, 5]. As such, anxiety-prone individuals detect random fluctuations in the bodily state that would normally remain sub-threshold, and then associate those signals with aversive or negative outcomes. In a complimentary but orthogonal literature, interoception has been conceptualized within a Bayesian predictive coding framework, whereby afferent interoceptive prediction errors update predictions about the condition of the body (e.g. [66, 67, 68]). Bayesian predictive coding models are guided by the free energy principle, which states that living organisms are motivated to reduce the difference between expected and encountered sensory inputs [69]. In predictive coding terms, this is actualized by having beliefs (or ‘priors’) about the world which guide predictions about incoming sensory input. Any differences between expected and encountered sensory input is represented as prediction error. Importantly, the relative weight or importance that we attribute to prediction errors is called ‘precision’. It is argued that precision is represented physiologically as post-synaptic gain of superficial pyramidal cells (which signal prediction error) [70, 71].

Within such a framework, altered beliefs that focus on the negative consequences of interoceptive states can be conceptualized as negatively biased priors. Similarly, a reduced afferent signal to noise ratio in anxiety can be likened to increased precision or synaptic gain on afferent interoceptive information. As suggested by Paulus and Stein [4, 5], these two mechanisms may work in tandem to increase the risk of anxiety. Specifically, in predictive coding terms, increased precision increases the weighting of prediction error signals, exacerbating the influence of biased priors on explaining the causes of the perceived sensations. The present findings suggest that the development of incorrect priors may in part be due to alexithymia.

A growing body of evidence now implicates altered interoceptive processing in a number of clinical conditions, including autism [33, 34, 72], eating disorders [35, 36, 37, 39, 41], and substance dependence [43, 73]. Alexithymia is also often seen in higher levels in these conditions than in the general population. Future work should seek to disentangle the relationship between the processing of somatic sensations and their integration into emotional states in each of these diagnostic categories. Approaching interoception from a dimensional perspective, as proposed by Garfinkel and colleagues [13, 14], is likely to prove crucial in this endeavor.

A few limitations of the present investigation are worth noting. The measures used in this study all rely on the validity of self-report. In addition, strong conclusions about the directionality of the effects are difficult to draw, due to the correlational nature of the observations. That is, it is also feasible that individuals who suffer from anxiety become increasingly alexithymic and sensitive to interoceptive sensations. Future research employing a longitudinal or interventionist approach may be required to fully delineate this. If it is confirmed that individuals who display both high levels of interoceptive sensibility and alexithymia go on to develop anxiety, interventions that target the attribution of bodily sensations may prove valuable in reducing anxiety.

Here, we present evidence that high levels of interoceptive sensibility, in combination with alexithymia, heightens the risk for clinically significant trait anxiety. We suggest that a heightened sensibility for interoceptive signals, combined with a difficulty in attributing these sensations to emotions, leaves these sensations vulnerable to catastrophizing interpretation. Within a Bayesian predicative coding framework, heightened interoceptive sensibility can be likened to increased precision on afferent interoceptive signals. This is combination with negatively biased priors about interoceptive states, which we argue may be caused by alexithymia, increases the individual’s risk for anxiety. Interventions that target the accurate attribution and interpretation of bodily sensations may prove valuable in reducing anxiety.

## Supporting information

S1 Text Simplified awareness subscale of the Porges Body Perception Questionnaire. The scale was modified by simplifying the language and removing 6 items, which were deemed collapsible into other items. As an example of language simplification, item 3) ‘An urge to cough to clear my throat’ became ‘A need to cough to clear my throat’. As an example of collapsing, items 17) ‘A bloated feeling because of water retention’ and 24) ‘Stomach distension or bloatedness’ were deemed collapsible into one: ‘A swollen tummy’. Care was taken to ensure the integral meaning of the items was not altered. This modification was originally designed to make the questionnaire mare accessible for children, such that child and adult participants could complete the same measure in future research, and has been validated in a sample of participants aged 6 to 18 years old, showing good reliability [33].
(DOCX)

S1 Table Factor analysis of items. Component loadings of items in abbreviated version of Awareness subsection of Porges Body Perception Questionnaire (BPQ [10, 33]), Toronto Alexithymia Scale (TAS-20 [54]) and Trait Form of the State Trait Anxiety Inventory (STAI [53]). The highest factor loading for each item is given below. The rotation converged in 66 iterations on 21 factors. Factors associated with the BPQ are shown in yellow, the TAS-20 in green, and the STAI-Trait in blue. Items from the BPQ, the TAS-20 and the STAI-Trait all loaded onto separate factors. That is, no factor contained items from different questionnaires.
(DOCX)

S1 Data Data. (XLSX)

## References

[1] Kessler RC, Berglund P, Demler O, Jin R, Merikangas KR, Walters EE. Lifetime prevalence and age-of-onset distributions of DSM-IV disorders in the National Comorbidity Survey Replication. Arch Gen Psychiatry. 2005 6 1;62(6):593–602. 10.1001/archpsyc.62.6.593 15939837

[2] Craig AD. How do you feel? Interoception: the sense of the physiological condition of the body. Nature Rev Neurosci. 2002 8;3(8):655. 12154366 10.1038/nrn894

[3] Craig AD. Significance of the insula for the evolution of human awareness of feelings from the body. Ann N Y Acad Sci. 2011 4;1225(1):72–82. 21534994 10.1111/j.1749-6632.2011.05990.x

[4] Paulus MP, Stein MB. An insular view of anxiety. Biol Psychiatry. 2006 8 15;60(4):383–7. 10.1016/j.biopsych.2006.03.042 16780813

[5] Paulus MP, Stein MB. Interoception in anxiety and depression. Brain Struct Funct. 2010 6 1;214(5–6):451–63. 10.1007/s00429-010-0258-9 20490545 PMC2886901

[6] Rauch SL, Savage CR, Alpert NM, Fischman AJ, Jenike MA. The functional neuroanatomy of anxiety: a study of three disorders using positron emission tomography and symptom provocation. Biol Psychiatry. 1997 9 15;42(6):446–52. 10.1016/S0006-3223(97)00145-5 9285080

[7] Clark DM et al Misinterpretation of body sensations in panic disorder. J Consult Clin Psychol. 1997 4;65(2):203 9086683 10.1037//0022-006x.65.2.203

[8] Nemiah JC, Freyberger H, Sifneos PE. Alexithymia: a view of the psychosomatic process In: Hill OW, editor. Modern Trends in Psychosomatic Medicine. 3rd vol. Butterworths, London; 1976 pp.430–439.

[9] Mandler G, Mandler JM, Uviller ET. Autonomic feedback: The perception of autonomic activity. J Abnorm Soc Psychol. 1958 5;56(3):367. 10.1037/h0048083 13538604

[10] Porges S. Body perception questionnaire. Laboratory of Developmental Assessment, University of Maryland 1993.

[11] Schandry R. Heart beat perception and emotional experience. Psychophysiology. 1981 7;18(4):483–8. 7267933 10.1111/j.1469-8986.1981.tb02486.x

[12] Whitehead WE, Drescher VM, Heiman P, Blackwell B. Relation of heart rate control to heartbeat perception. Biofeedback Self Regul. 1977 12 1;2(4):371–92. 612350

[13] Garfinkel SN, Critchley HD. Interoception, emotion and brain: new insights link internal physiology to social behaviour. Commentary on: “Anterior insular cortex mediates bodily sensibility and social anxiety” by Terasawa et al.(2012). Soc Cogn Affect Neurosci. 2013 3 1;8(3):231–4. 10.1093/scan/nss140 23482658 PMC3594730

[14] Garfinkel SN, Seth AK, Barrett AB, Suzuki K, Critchley HD. Knowing your own heart: distinguishing interoceptive accuracy from interoceptive awareness. Biol Psychol. 2015 1 1;104:65–74. 10.1016/j.biopsycho.2014.11.004 25451381

[15] Pollatos O, Traut-Mattausch E, Schroeder H, Schandry R. Interoceptive awareness mediates the relationship between anxiety and the intensity of unpleasant feelings. J Anxiety Disord. 2007 1 1;21(7):931–43. 10.1016/j.janxdis.2006.12.004 17257810

[16] Dunn BD, Stefanovitch I, Evans D, Oliver C, Hawkins A, Dalgleish T. Can you feel the beat? Interoceptive awareness is an interactive function of anxiety-and depression-specific symptom dimensions. Behav Res Ther. 2010 11 1;48(11):1133–8. 10.1016/j.brat.2010.07.006 20692645 PMC2964892

[17] Stevens S, Gerlach AL, Cludius B, Silkens A, Craske MG, Hermann C. Heartbeat perception in social anxiety before and during speech anticipation. Behav Res Ther. 2011 2 1;49(2):138–43. 10.1016/j.brat.2010.11.009 21147477

[18] Ehlers A, Margraf J, Roth WT, Taylor CB, Birbaumer N. Anxiety induced by false heart rate feedback in patients with panic disorder. Behav Res Ther. 1988 1 1;26(1):1–1. 3341996 10.1016/0005-7967(88)90028-9

[19] De Pascalis V, Alberti ML, Pandolfo R. Anxiety, perception, and control of heart rate. Percept Mot Skills. 1984 8;59(1):203–11. 10.2466/pms.1984.59.1.203 6493936

[20] De Berardis D et al Alexithymia, fear of bodily sensations, and somatosensory amplification in young outpatients with panic disorder. Psychosomatics. 2007 5 1;48(3):239–46. 10.1176/appi.psy.48.3.239 17478593

[21] Olatunji BO, Deacon BJ, Abramowitz JS, Valentiner DP. Body vigilance in nonclinical and anxiety disorder samples: structure, correlates, and prediction of health concerns. Behav Ther. 2007 12 1;38(4):392–401. 10.1016/j.beth.2006.09.002 18021953

[22] Gregor KL, Zvolensky MJ. Anxiety sensitivity and perceived control over anxiety-related events: Evaluating the singular and interactive effects in the prediction of anxious and fearful responding to bodily sensations. Behav Res Ther. 2008 9 1;46(9):1017–25. 10.1016/j.brat.2008.06.003 18675954

[23] Anderson ER, Hope DA. The relationship among social phobia, objective and perceived physiological reactivity, and anxiety sensitivity in an adolescent population. J Anxiety Disord. 2009 1 1;23(1):18–26. 10.1016/j.janxdis.2008.03.011 18436426 PMC2645715

[24] Chambless DL, Caputo GC, Bright P, Gallagher R. Assessment of fear of fear in agoraphobics: the body sensations questionnaire and the agoraphobic cognitions questionnaire. J Consult Clin Psychol 1984 12;52(6):1090 6520279 10.1037//0022-006x.52.6.1090

[25] Schmidt NB, Lerew DR, Trakowski JH. Body vigilance in panic disorder: Evaluating attention to bodily perturbations. J Consult Clin Psychol. 1997 4;65(2):214 9086684 10.1037//0022-006x.65.2.214

[26] Salminen JK, Saarijärvi S, Äärelä E, Toikka T, Kauhanen J. Prevalence of alexithymia and its association with sociodemographic variables in the general population of Finland. J Psychosom Res. 1999 1 1;46(1):75–82. 10088984 10.1016/s0022-3999(98)00053-1

[27] Besharat MA. Relations between alexithymia, anxiety, depression, psychological distress, and psychological well-being. J Psychol. 2008 3(10): 17–40.

[28] Devine H, Stewart SH, Watt MC. Relations between anxiety sensitivity and dimensions of alexithymia in a young adult sample. J Psychosom Res. 1999 8 1;47(2):145–58. 10579498 10.1016/s0022-3999(99)00033-1

[29] Herbert BM, Herbert C, Pollatos O. On the relationship between interoceptive awareness and alexithymia: is interoceptive awareness related to emotional awareness?. J Pers. 2011 10;79(5):1149–75. 10.1111/j.1467-6494.2011.00717.x 21241306

[30] Griffin C, Lombardo MV, Auyeung B. Alexithymia in children with and without autism spectrum disorders. Autism Res. 2016 7;9(7):773–80. 10.1002/aur.1569 26426084

[31] Milosavljevic B et al Alexithymia in adolescents with autism spectrum disorder: its relationship to internalising difficulties, sensory modulation and social cognition. J Autism Dev Disord. 2016 4 1;46(4):1354–67. 10.1007/s10803-015-2670-8 26659552

[32] Hill E, Berthoz S, Frith U. Brief report: Cognitive processing of own emotions in individuals with autistic spectrum disorder and in their relatives. J Autism Dev Disord. 2004 4 1;34(2):229–35. 15162941 10.1023/b:jadd.0000022613.41399.14

[33] Palser ER, Fotopoulou A, Pellicano E, Kilner JM. The link between interoceptive processing and anxiety in children diagnosed with autism spectrum disorder: Extending adult findings into a developmental sample. Biol Psychol. 2018 7 1;136:13–21. 10.1016/j.biopsycho.2018.05.003 29742462

[34] Garfinkel SN, Tiley C, O'Keeffe S, Harrison NA, Seth AK, Critchley HD. Discrepancies between dimensions of interoception in autism: Implications for emotion and anxiety. Biol Psychol. 2016 2 1;114:117–26. 10.1016/j.biopsycho.2015.12.003 26724504

[35] Berner LA, Simmons AN, Wierenga CE, Bischoff-Grethe A, Paulus MP, Bailer UF, Ely AV, Kaye WH. Altered interoceptive activation before, during, and after aversive breathing load in women remitted from anorexia nervosa. Psychol Med. 2018 1;48(1):142–54. 10.1017/S0033291717001635 28714434 PMC5990016

[36] Khalsa SS, Craske MG, Li W, Vangala S, Strober M, Feusner JD. Altered interoceptive awareness in anorexia nervosa: effects of meal anticipation, consumption and bodily arousal. Int J Eat Disord. 2015 11;48(7):889–97. 10.1002/eat.22387 25712775 PMC4898968

[37] Klabunde M, Acheson DT, Boutelle KN, Matthews SC, Kaye WH. Interoceptive sensitivity deficits in women recovered from bulimia nervosa. Eat Behav. 2013 12 1;14(4):488–92. 10.1016/j.eatbeh.2013.08.002 24183142 PMC3817494

[38] Montebarocci O, Codispoti M, Surcinelli P, Franzoni E, Baldaro B, Rossi N. Alexithymia in female patients with eating disorders. Eat Weight Disord. 2006 3 1;11(1):14–21. 16801741 10.1007/BF03327739

[39] Pollatos O et al Reduced perception of bodily signals in anorexia nervosa. Eat Behav. 2008 12 1;9(4):381–8. 10.1016/j.eatbeh.2008.02.001 18928900

[40] Swinbourne JM, Touyz SW. The co‐morbidity of eating disorders and anxiety disorders: A review. Eur Eat Disord Rev. 2007 7;15(4):253–74. 10.1002/erv.784 17676696

[41] Fassino S, Pierò A, Gramaglia C, Abbate-Daga G. Clinical, psychopathological and personality correlates of interoceptive awareness in anorexia nervosa, bulimia nervosa and obesity. Psychopathology. 2004;37(4):168–74. 10.1159/000079420 15237246

[42] Cox BJ, Norton GR, Swinson RP, Endler NS. Substance abuse and panic-related anxiety: a critical review. Behav Res Ther. 1990 1 1;28(5):385–93. 2256896 10.1016/0005-7967(90)90157-e

[43] Naqvi NH, Bechara A. The insula and drug addiction: an interoceptive view of pleasure, urges, and decision-making. Brain Struct Funct. 2010 6 1;214(5–6):435–50. 10.1007/s00429-010-0268-7 20512364 PMC3698865

[44] Paulus MP, Stewart JL. Interoception and drug addiction. Neuropharmacol. 2014 1 1;76:342–50. 10.1016/j.neuropharm.2013.07.002 PMC3858461 23855999

[45] Gurney C, Roth M, Garside RF, Kerr TA, Schapira K. Studies in the classification of affective disorders: The relationship between anxiety states and depressive illnesses—II. Br J Psychiatry. 1972 8;121(561):162–6. 5072241 10.1192/bjp.121.2.162

[46] Simmons WK et al Depression-related increases and decreases in appetite: dissociable patterns of aberrant activity in reward and interoceptive neurocircuitry. Am J Psychiatry. 2016 1 22;173(4):418–28. 10.1176/appi.ajp.2015.15020162 26806872 PMC4818200

[47] Murphy J, Catmur C, Bird G. Alexithymia is associated with a multidomain, multidimensional failure of interoception: Evidence from novel tests. J Exp Psychol Gen. 2018 3;147(3):398 10.1037/xge0000366 29154612 PMC5824617

[48] Shah P, Hall R, Catmur C, Bird G. Alexithymia, not autism, is associated with impaired interoception. Cortex. 2016 8 1;81:215–20. 10.1016/j.cortex.2016.03.021 27253723 PMC4962768

[49] Nicholson TM, Williams DM, Grainger C, Christensen JF, Calvo-Merino B, Gaigg SB. Interoceptive impairments do not lie at the heart of autism or alexithymia. J Abnorm Psychol. 2018 8;127(6):612 10.1037/abn0000370 30102067 PMC6089261

[50] Zamariola G, Vlemincx E, Luminet O, Corneille O. Relationship between interoceptive accuracy, interoceptive sensibility, and alexithymia. Pers Individ Dif. 2018 4 15;125:14–20.

[51] Nakao M, Barsky AJ, Kumano H, Kuboki T. Relationship between somatosensory amplification and alexithymia in a Japanese psychosomatic clinic. Psychosomatics. 2002 1 1;43(1):55–60. 10.1176/appi.psy.43.1.55 11927759

[52] Longarzo M, D'Olimpio F, Chiavazzo A, Santangelo G, Trojano L, Grossi D. The relationships between interoception and alexithymic trait. The Self-Awareness Questionnaire in healthy subjects. Frontiers Psychol. 2015 8 7;6:1149. 10.3389/fpsyg.2015.01149 PMC4528101 26300829

[53] Betka S et al How Do Self‐Assessment of Alexithymia and Sensitivity to Bodily Sensations Relate to Alcohol Consumption?. Alcohol Clin Exp Res. 2018 1;42(1):81–8. 10.1111/acer.13542 29094768

[54] Spielberger CD, Gorsuch RL, Lushene RE, Vagg PR, Jacobs GA. State-trait anxiety inventory. Palo Alto.

[55] Taylor GJ, Ryan D, Bagby M. Toward the development of a new self-report alexithymia scale. Psychother Psychosom. 1985;44(4):191–9. 10.1159/000287912 3837277

[56] Baron RM, Kenny DA. The moderator–mediator variable distinction in social psychological research: Conceptual, strategic, and statistical considerations. Pers Soc Psychol. 1986 12;51(6):1173. 10.1037//0022-3514.51.6.1173 3806354

[57] American Psychiatric Association. Diagnostic and statistical manual of mental disorders (DSM-5®). American Psychiatric Pub; 2013 5 22.

[58] Livingston LA, Livingston LM. Commentary: alexithymia, not autism, is associated with impaired interoception. Front Psychol. 2016 7 22;7:1103 10.3389/fpsyg.2016.01103 27501009 PMC4956654

[59] Mul CL, Stagg SD, Herbelin B, Aspell JE. The Feeling of Me Feeling for You: Interoception, Alexithymia and Empathy in Autism. J Autism Dev Disord. 2018 4 11:1–5. 10.1007/s10803-018-3564-3 29644587

[60] Murphy J, Brewer R, Hobson H, Catmur C, Bird G. Is alexithymia characterised by impaired interoception? Further evidence, the importance of control variables, and the problems with the Heartbeat Counting Task. Biol Psychol. 2018 5 24. 10.1016/j.biopsycho.2018.05.010 29803614

[61] Mehling W. Differentiating attention styles and regulatory aspects of self-reported interoceptive sensibility. Phil Trans R Soc B. 2016 11 19;371(1708):20160013 10.1098/rstb.2016.0013 28080970 PMC5062101

[62] Mehling WE, Price C, Daubenmier JJ, Acree M, Bartmess E, Stewart A. The multidimensional assessment of interoceptive awareness (MAIA). PloS one. 2012 11 1;7(11):e48230 10.1371/journal.pone.0048230 23133619 PMC3486814

[63] Muir K, Madill A, Brown C. Individual differences in emotional processing and autobiographical memory: interoceptive awareness and alexithymia in the fading affect bias. Cogn Emot. 2017 10 3;31(7):1392–404. 10.1080/02699931.2016.1225005 27556549

[64] Brewer R, Cook R, Bird G. Alexithymia: a general deficit of interoception. R Soc Open Sci 2016 10 1;3(10):150664 10.1098/rsos.150664 27853532 PMC5098957

[65] Domschke K, Stevens S, Pfleiderer B, Gerlach AL. Interoceptive sensitivity in anxiety and anxiety disorders: an overview and integration of neurobiological findings. Clin Psychol Rev. 2010 2 1;30(1):1–1. 10.1016/j.cpr.2009.08.008 19751958

[66] Ainley V, Apps MA, Fotopoulou A, Tsakiris M. ‘Bodily precision’: a predictive coding account of individual differences in interoceptive accuracy. Phil Trans R Soc B 2016 11 19;371(1708):20160003 10.1098/rstb.2016.0003 28080962 PMC5062093

[67] Fotopoulou A. Beyond the reward principle: consciousness as precision seeking. Neuropsychoanalysis. 2013 1 1;15(1):33–8.

[68] Pezzulo G, Rigoli F, Friston K. Active Inference, homeostatic regulation and adaptive behavioural control. Prog Neurobiol. 2015 11 1;134:17–35. 10.1016/j.pneurobio.2015.09.001 26365173 PMC4779150

[69] Friston K. The free-energy principle: a rough guide to the brain?. Trends Cogn Sci. 2009 7 1;13(7):293–301. 10.1016/j.tics.2009.04.005 19559644

[70] Feldman H, Friston K. Attention, uncertainty, and free-energy. Front Hum Neurosci. 2010 12 2;4:215 10.3389/fnhum.2010.00215 21160551 PMC3001758

[71] Shipp S, Adams RA, Friston KJ. Reflections on agranular architecture: predictive coding in the motor cortex. Trends Neurosci. 2013 12 1;36(12):706–16. 10.1016/j.tins.2013.09.004 24157198 PMC3858810

[72] Quattrocki E, Friston K. Autism, oxytocin and interoception. Neurosci Biobehav Rev. 2014 11 1;47:410–30. 10.1016/j.neubiorev.2014.09.012 25277283 PMC4726659

[73] Verdejo-Garcia A, Clark L, Dunn BD. The role of interoception in addiction: a critical review. Neuroscience & Biobehavioral Reviews. 2012 9 1;36(8):1857–69. 22659642 10.1016/j.neubiorev.2012.05.007

