# Supplementary material for: Alexithymia mediates the relationship between interoceptive sensibility and anxiety
Source: PLoS One. 2018 Sep 13;13(9):e0203212. doi: 10.1371/journal.pone.0203212 (PMC6136731; doi:10.1371/journal.pone.0203212)
Supplement: S1 Text — The scale was modified by simplifying the language and removing 6 items, which were deemed collapsible into other items. As an example of language simplification, item 3) ‘An urge to cough to clear my throat’ became ‘A need to cough to clear my throat’. As an example of collapsing, items 17) ‘A bloated feeling because of water retention’ and 24) ‘Stomach distension or bloatedness’ were deemed collapsible into one: ‘A swollen tummy’. Care was taken to ensure the integral meaning of the items was not altered. This modification was originally designed to make the questionnaire mare accessible for children, such that child and adult participants could complete the same measure in future research, and has been validated in a sample of participants aged 6 to 18 years old, showing good reliability [33]. (DOCX) [file pone.0203212.s001.docx]

## S1 Text: Simplified Awareness Subscale of the Body Perception Questionnaire

**I: Awareness**

Imagine how you feel your body processes. Draw a circle around the answer that sounds most like you. Answer how often you feel the things below:

Most of the time I can feel myself:

1. **Swallowing a lot**

Never Occasionally Sometimes Usually Always

1. **Ringing in my ears**

Never Occasionally Sometimes Usually Always

1. **A need to cough to clear my throat**

Never Occasionally Sometimes Usually Always

1. **My body swaying when I am standing**

Never Occasionally Sometimes Usually Always

1. **My mouth being dry**

Never Occasionally Sometimes Usually Always

1. **How fast I am breathing**

Never Occasionally Sometimes Usually Always

1. **Watery eyes**

Never Occasionally Sometimes Usually Always

1. **My skin itching**

Never Occasionally Sometimes Usually Always

1. **Noises in my stomach after I’ve eaten**

Never Occasionally Sometimes Usually Always

1. **Tired or painful eyes**

Never Occasionally Sometimes Usually Always

1. **An ache in my neck or back**

Never Occasionally Sometimes Usually Always

1. **Swelling in my body or parts of my body**

Never Occasionally Sometimes Usually Always

1. **Need to go to the toilet (wee)**

Never Occasionally Sometimes Usually Always

1. **Shaky hands**

Never Occasionally Sometimes Usually Always

1. **Need to go to the toilet (poo)**

Never Occasionally Sometimes Usually Always

1. **An ache in my arms or legs**

Never Occasionally Sometimes Usually Always

1. **A swollen tummy**

Never Occasionally Sometimes Usually Always

1. **An ache in my face**

Never Occasionally Sometimes Usually Always

1. **Goose bumps**

Never Occasionally Sometimes Usually Always

1. **Twitchy face**

Never Occasionally Sometimes Usually Always

1. **Really tired**

Never Occasionally Sometimes Usually Always

1. **Tummy pain**

Never Occasionally Sometimes Usually Always

1. **Fluttery eyes**

Never Occasionally Sometimes Usually Always

1. **Sweaty hands**

Never Occasionally Sometimes Usually Always

1. **Sweaty forehead**

Never Occasionally Sometimes Usually Always

1. **Being clumsy and bumping into people**

Never Occasionally Sometimes Usually Always

1. **Shaky lips**

Never Occasionally Sometimes Usually Always

1. **Sweaty armpits**

Never Occasionally Sometimes Usually Always

1. **Prickly skin, tingly skin, or numb skin**

Never Occasionally Sometimes Usually Always

1. **A hot or cold face (especially ears)**

Never Occasionally Sometimes Usually Always

1. **Grinding my teeth**

Never Occasionally Sometimes Usually Always

1. **Can’t be still**

Never Occasionally Sometimes Usually Always

1. **My eyes moving**

Never Occasionally Sometimes Usually Always

1. **Itchy nose**

Never Occasionally Sometimes Usually Always

1. **The hair on the back of my neck standing up**

Never Occasionally Sometimes Usually Always

1. **Needing a rest**

Never Occasionally Sometimes Usually Always

1. **Can’t focus my eyes**

Never Occasionally Sometimes Usually Always

1. **How hard my heart is beating**

Never Occasionally Sometimes Usually Always

1. **Feeling like I can’t go to the toilet when I try**

Never Occasionally Sometimes Usually Always

## Reference

1 Palser ER, Fotopoulou A, Pellicano E, Kilner JM. The link between interoceptive processing and anxiety in children diagnosed with autism spectrum disorder: Extending adult findings into a developmental sample. Biol Psychol. 2018 Jul 1;136:13-21.
