# Supplementary material for: Alexithymia mediates the relationship between interoceptive sensibility and anxiety
Source: PLoS One. 2018 Sep 13;13(9):e0203212. doi: 10.1371/journal.pone.0203212 (PMC6136731; doi:10.1371/journal.pone.0203212)
Supplement: S1 Table — Component loadings of items in abbreviated version of Awareness subsection of Porges Body Perception Questionnaire (BPQ [10, 33]), Toronto Alexithymia Scale (TAS-20 [54]) and Trait Form of the State Trait Anxiety Inventory (STAI [53]). The highest factor loading for each item is given below. The rotation converged in 66 iterations on 21 factors. Factors associated with the BPQ are shown in yellow, the TAS-20 in green, and the STAI-Trait in blue. Items from the BPQ, the TAS-20 and the STAI-Trait all loaded onto separate factors. That is, no factor contained items from different questionnaires. (DOCX) [file pone.0203212.s002.docx]

S1 Table: Factor analysis of items

| **ITEM** | **FACTOR** | | | | | | | | | | | | | | | | | | | |
| --- | --- | --- | --- | --- | --- | --- | --- | --- | --- | --- | --- | --- | --- | --- | --- | --- | --- | --- | --- | --- |
|  | **1** | **2** | **3** | **4** | **5** | **6** | **7** | **8** | **9** | **10** | **11** | **12** | **13** | **14** | **15** | **16** | **17** | **18** | **19** | **20** |
| BPQ_1 |  |  |  |  |  |  |  |  |  |  |  |  |  | .53 |  |  |  |  |  |  |
| BPQ_2 |  |  |  |  |  |  |  |  |  |  |  |  |  |  |  |  |  | .47 |  |  |
| BPQ_3 | .27 |  |  |  |  |  |  |  |  |  |  |  |  |  |  |  |  |  |  |  |
| BPQ_4 | .25 |  |  |  |  |  |  |  |  |  |  |  |  |  |  |  |  |  |  |  |
| BPQ_5 | .36 |  |  |  |  |  |  |  |  |  |  |  |  |  |  |  |  |  |  |  |
| BPQ_6 |  |  |  |  |  |  |  |  |  | .57 |  |  |  |  |  |  |  |  |  |  |
| BPQ_7 | .56 |  |  |  |  |  |  |  |  |  |  |  |  |  |  |  |  |  |  |  |
| BPQ_8 | .56 |  |  |  |  |  |  |  |  |  |  |  |  |  |  |  |  |  |  |  |
| BPQ_9 | .32 |  |  |  |  |  |  |  |  |  |  |  |  |  |  |  |  |  |  |  |
| BPQ_10 | .62 |  |  |  |  |  |  |  |  |  |  |  |  |  |  |  |  |  |  |  |
| BPQ_11 | .56 |  |  |  |  |  |  |  |  |  |  |  |  |  |  |  |  |  |  |  |
| BPQ_12 | .53 |  |  |  |  |  |  |  |  |  |  |  |  |  |  |  |  |  |  |  |
| BPQ_13 |  |  |  |  |  |  |  | .80 |  |  |  |  |  |  |  |  |  |  |  |  |
| BPQ_14 | .42 |  |  |  |  |  |  |  |  |  |  |  |  |  |  |  |  |  |  |  |
| BPQ_15 |  |  |  |  |  |  |  | .73 |  |  |  |  |  |  |  |  |  |  |  |  |
| BPQ_16 | .56 |  |  |  |  |  |  |  |  |  |  |  |  |  |  |  |  |  |  |  |
| BPQ_17 | .48 |  |  |  |  |  |  |  |  |  |  |  |  |  |  |  |  |  |  |  |
| BPQ_18 | .67 |  |  |  |  |  |  |  |  |  |  |  |  |  |  |  |  |  |  |  |
| BPQ_19 | .61 |  |  |  |  |  |  |  |  |  |  |  |  |  |  |  |  |  |  |  |
| BPQ_20 | .59 |  |  |  |  |  |  |  |  |  |  |  |  |  |  |  |  |  |  |  |
| BPQ_21 | .47 |  |  |  |  |  |  |  |  |  |  |  |  |  |  |  |  |  |  |  |
| BPQ_22 | .64 |  |  |  |  |  |  |  |  |  |  |  |  |  |  |  |  |  |  |  |
| BPQ_23 | .51 |  |  |  |  |  |  |  |  |  |  |  |  |  |  |  |  |  |  |  |
| BPQ_24 |  |  |  |  |  | .63 |  |  |  |  |  |  |  |  |  |  |  |  |  |  |
| **ITEM** | **1** | **2** | **3** | **4** | **5** | **6** | **7** | **8** | **9** | **10** | **11** | **12** | **13** | **14** | **15** | **16** | **17** | **18** | **19** | **20** |
| BPQ_25 |  |  |  |  |  | .59 |  |  |  |  |  |  |  |  |  |  |  |  |  |  |
| BPQ_26 | .40 |  |  |  |  |  |  |  |  |  |  |  |  |  |  |  |  |  |  |  |
| BPQ_27 | .43 |  |  |  |  |  |  |  |  |  |  |  |  |  |  |  |  |  |  |  |
| BPQ_28 |  |  |  |  |  | .54 |  |  |  |  |  |  |  |  |  |  |  |  |  |  |
| BPQ_29 | .54 |  |  |  |  |  |  |  |  |  |  |  |  |  |  |  |  |  |  |  |
| BPQ_30 | .40 |  |  |  |  |  |  |  |  |  |  |  |  |  |  |  |  |  |  |  |
| BPQ_31 |  |  |  |  |  |  |  |  |  |  |  |  |  |  |  |  |  |  | .31 |  |
| BPQ_32 |  |  |  |  |  |  | .40 |  |  |  |  |  |  |  |  |  |  |  |  |  |
| BPQ_33 |  |  |  |  |  |  | .60 |  |  |  |  |  |  |  |  |  |  |  |  |  |
| BPQ_34 | .47 |  |  |  |  |  |  |  |  |  |  |  |  |  |  |  |  |  |  |  |
| BPQ_35 | .42 |  |  |  |  |  |  |  |  |  |  |  |  |  |  |  |  |  |  |  |
| BPQ_36 |  |  |  |  |  |  |  |  |  |  |  |  |  |  | .62 |  |  |  |  |  |
| BPQ_37 | .45 |  |  |  |  |  |  |  |  |  |  |  |  |  |  |  |  |  |  |  |
| BPQ_38 |  |  |  |  |  |  |  |  |  | .60 |  |  |  |  |  |  |  |  |  |  |
| BPQ_39 | .40 |  |  |  |  |  |  |  |  |  |  |  |  |  |  |  |  |  |  |  |
| TAS_1 |  |  | .73 |  |  |  |  |  |  |  |  |  |  |  |  |  |  |  |  |  |
| TAS_2 |  |  | .71 |  |  |  |  |  |  |  |  |  |  |  |  |  |  |  |  |  |
| TAS_3 |  |  |  |  |  |  |  |  |  |  | .45 |  |  |  |  |  |  |  |  |  |
| TAS_4 |  |  | .52 |  |  |  |  |  |  |  |  |  |  |  |  |  |  |  |  |  |
| TAS_5 |  |  |  |  |  |  |  |  |  |  |  |  | .59 |  |  |  |  |  |  |  |
| TAS_6 |  |  | .57 |  |  |  |  |  |  |  |  |  |  |  |  |  |  |  |  |  |
| TAS_7 |  |  |  |  |  |  |  |  |  |  | .48 |  |  |  |  |  |  |  |  |  |
| TAS_8 |  |  |  |  |  |  |  |  |  |  |  |  |  |  |  | .30 |  |  |  |  |
| TAS_9 |  |  | .71 |  |  |  |  |  |  |  |  |  |  |  |  |  |  |  |  |  |
| TAS_10 |  |  |  |  | .65 |  |  |  |  |  |  |  |  |  |  |  |  |  |  |  |
| TAS_11 |  |  | .65 |  |  |  |  |  |  |  |  |  |  |  |  |  |  |  |  |  |
| TAS_12 |  |  | .44 |  |  |  |  |  |  |  |  |  |  |  |  |  |  |  |  |  |
| TAS_13 |  |  | .57 |  |  |  |  |  |  |  |  |  |  |  |  |  |  |  |  |  |
| **ITEM** | **1** | **2** | **3** | **4** | **5** | **6** | **7** | **8** | **9** | **10** | **11** | **12** | **13** | **14** | **15** | **16** | **17** | **18** | **19** | **20** |
| TAS_14 |  |  | .44 |  |  |  |  |  |  |  |  |  |  |  |  |  |  |  |  |  |
| TAS_15 |  |  |  |  | .61 |  |  |  |  |  |  |  |  |  |  |  |  |  |  |  |
| TAS_16 |  |  |  |  |  |  |  |  |  |  |  |  |  |  |  | .44 |  |  |  |  |
| TAS_17 |  |  |  |  | .45 |  |  |  |  |  |  |  |  |  |  |  |  |  |  |  |
| TAS_18 |  |  |  |  |  |  |  |  |  |  |  |  | .32 |  |  |  |  |  |  |  |
| TAS_19 |  |  |  |  |  |  |  |  |  |  |  |  | .26 |  |  |  |  |  |  |  |
| TAS_20 |  |  |  |  |  |  |  |  |  |  |  |  |  |  |  | .41 |  |  |  |  |
| STAI_21 |  | .64 |  |  |  |  |  |  |  |  |  |  |  |  |  |  |  |  |  |  |
| STAI_22 |  | .44 |  |  |  |  |  |  |  |  |  |  |  |  |  |  |  |  |  |  |
| STAI_23 |  | .66 |  |  |  |  |  |  |  |  |  |  |  |  |  |  |  |  |  |  |
| STAI_24 |  | .43 |  |  |  |  |  |  |  |  |  |  |  |  |  |  |  |  |  |  |
| STAI_25 |  |  |  |  |  |  |  |  | .55 |  |  |  |  |  |  |  |  |  |  |  |
| STAI_26 |  | .49 |  |  |  |  |  |  |  |  |  |  |  |  |  |  |  |  |  |  |
| STAI_27 |  | .58 |  |  |  |  |  |  |  |  |  |  |  |  |  |  |  |  |  |  |
| STAI_28 |  |  |  | .45 |  |  |  |  |  |  |  |  |  |  |  |  |  |  |  |  |
| STAI_29 |  |  |  | .64 |  |  |  |  |  |  |  |  |  |  |  |  |  |  |  |  |
| STAI_30 |  |  |  | .75 |  |  |  |  |  |  |  |  |  |  |  |  |  |  |  |  |
| STAI_31 |  |  |  | .33 |  |  |  |  |  |  |  |  |  |  |  |  |  |  |  |  |
| STAI_32 |  |  |  |  |  |  |  |  |  |  |  | .47 |  |  |  |  |  |  |  |  |
| STAI_33 |  | .71 |  |  |  |  |  |  |  |  |  |  |  |  |  |  |  |  |  |  |
| STAI_34 |  |  |  |  |  |  |  |  |  |  |  | .41 |  |  |  |  |  |  |  |  |
| STAI_35 |  |  |  |  |  |  |  |  | .64 |  |  |  |  |  |  |  |  |  |  |  |
| STAI_36 |  | .71 |  |  |  |  |  |  |  |  |  |  |  |  |  |  |  |  |  |  |
| STAI_37 |  |  |  | .57 |  |  |  |  |  |  |  |  |  |  |  |  |  |  |  |  |
| STAI_38 |  |  |  | .56 |  |  |  |  |  |  |  |  |  |  |  |  |  |  |  |  |
| STAI_39 |  | .62 |  |  |  |  |  |  |  |  |  |  |  |  |  |  |  |  |  |  |
| STAI_40 |  |  |  | .63 |  |  |  |  |  |  |  |  |  |  |  |  |  |  |  |  |
